# Case report: Advanced primary squamous cell carcinoma in the periampullary area with upregulation of programmed cell death-ligand 1 expression and response to sintilimab immunotherapy

**DOI:** 10.3389/fimmu.2023.1086760

**Published:** 2023-01-27

**Authors:** Baoshan Wang, Dazhou Li, Dehua Zeng, Wen Wang, Chuanshen Jiang

**Affiliations:** ^1^ Department of Gastroenterology, 900^TH^Hospital of Joint Logistics Support Force, Fujian Medical University, Fuzhou, China; ^2^ Department of Pathology, 900^TH^Hospital of Joint Logistics Support Force, Fujian Medical University, Fuzhou, China; ^3^ Department of Gastroenterology, 900^TH^Hospital of Joint Logistics Support Force, Oriental Hospital Affiliated to Xiamen University, Fuzhou, China

**Keywords:** periampullary, programmed cell death-ligand 1, primary, immunotherapy, sintilimab, squamous cell carcinoma

## Abstract

Primary squamous cell carcinoma (SCC) of the periampullary area is exceedingly rare. We report a case of a 45-year-old woman who presented with progressive upper abdominal pain and melena. Laboratory testing revealed an elevated level of carcinoembryonic antigen. Esophagogastroduodenoscopy revealed a very large irregular ulcerated tumor in the periampullary area. Contrast-enhanced computed tomography (CT) of the chest, abdomen, and pelvis, 18 F-fluorodeoxyglucose positron emission tomography/CT, and thin-prep cytologic test excluded metastasis of the primary tumor to the periampullary area from other sites. Immunohistochemistry revealed positive p40 and cytokeratin (CK)5/6, indicating SCC. The expression of programmed cell death-ligand 1 (PD-L1) in tumor cells was upregulated, and the patient responded well to chemotherapy combined with immunotherapy. To the best of our knowledge, this is the first reported case of advanced primary SCC in the periampullary area with high expression of PD-L1.

## 1 Introduction

Periampullary cancers, including common bile duct, pancreatic ductal, ampulla of Vater, and duodenal cancers, are highly lethal malignancies with poor prognoses, with less than 5% of patients achieving the overall median 5-year survival rate (1). The majority of these tumors are adenocarcinomas, and primary SCC is exceedingly rare, with only occasional cases reported in the literature. Thus far, no clinical guidelines or standard treatment approaches are available because of the rarity of SCC of the periampullary area. It has recently been reported that the use of pembrolizumab, a programmed cell death protein-1 monoclonal antibody, produced a durable response in a patient with metastatic disease after surgery and chemotherapy for PD-L1-positive ampullary carcinoma (2). Here, we report on a case of a patient having advanced primary SCC of the periampullary area with upregulation of PD-L1 expression in tumor cells who responded well to chemotherapy combined with immunotherapy. To our knowledge, this is the first reported case of advanced primary SCC of the periampullary area with high PD-L1 expression.

## 2 Case description

A 45-year-old woman was referred to our department on May 8, 2022, with a 1-month history of progressive upper abdominal pain and melena. Her epigastric pain appeared on an empty stomach, was relieved after meals, and was accompanied by the voiding of unformed black stools 2–3 times per day, with the stool volume being approximately 50 ml each time. Other associated symptoms included unintentional weight loss and fatigue. Her physical examination revealed anemia with pale conjunctiva. She also exhibited epigastric tenderness without rebound tenderness and involuntary guarding. Moreover, a hard, painless but moveable lump approximately 5 × 5 cm in size was noted in the upper abdomen. Laboratory tests revealed a carcinoembryonic antigen (CEA) level of 29.43 ng/ml but normal levels of carbohydrate antigen (CA)19-9, CA125, and alpha-fetoprotein.

**Figure 1:**
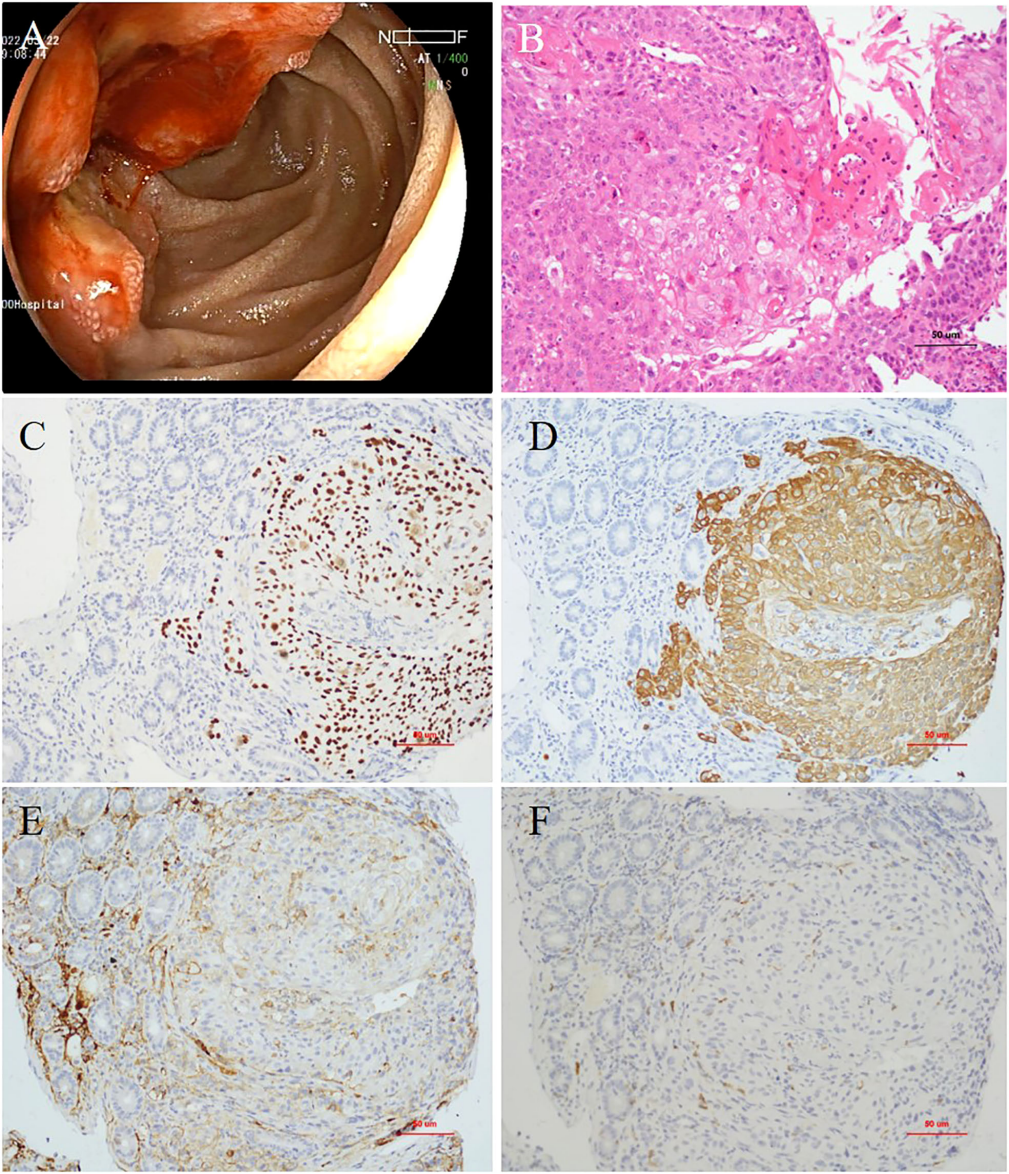
**(A)** Esophagogastroduodenoscopy showing a very large irregular ulcerated tumor in the periampullary area. **(B)** Histopathology showing invasive moderately differentiated squamous cell carcinoma (hematoxylin and eosin staining, ×200 magnification). **(C)** Expression of P40 (×200 magnification) and **(D)** cytokeratin (CK)5/6 detected by immunohistochemistry (×200 magnification). **(E)** Expression of programmed cell death-ligand 1 (PD-L1; ×200 magnification) and **(F)** programmed cell death protein-1 (PD-1) detected by immunohistochemistry (×200 magnification).

**Figure 2:**
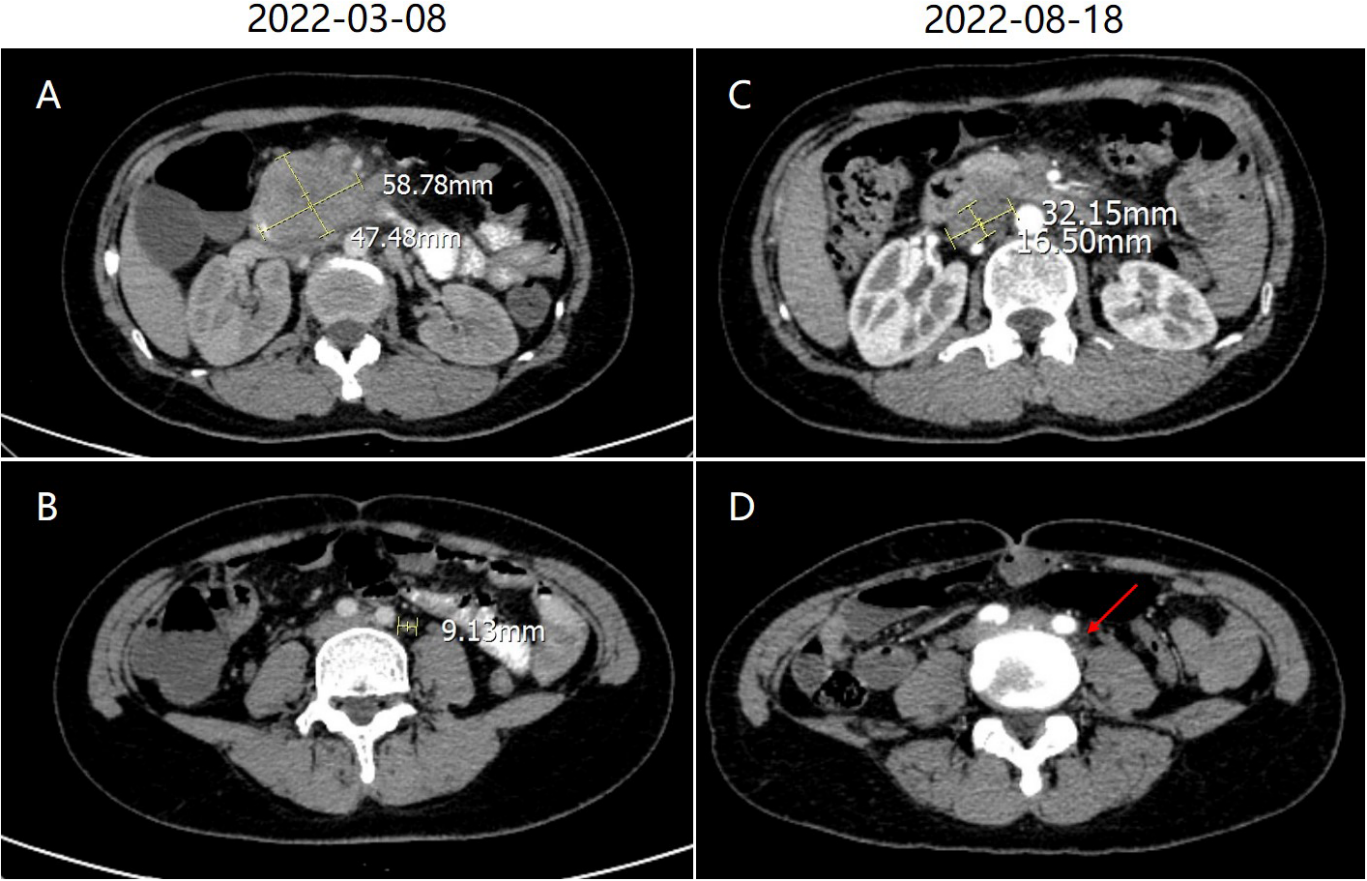
**(A)** Abdominal contrast-enhanced computed tomography (CT) showed a mass approximately 5.8 × 4.7 cm in size in the periampullary area. **(B)** The largest lymph node in the retroperitoneal space was approximately 1.0 cm in size. **(C)** After treatment, abdominal enhanced CT showed shrinkage of the primary lesion. **(D)** The largest lymph node also shrank (red arrow).

Esophagogastroduodenoscopy revealed a very large irregular ulcerated tumor with volcano-like changes around it in the periampullary area (**Figure 1A**). Six biopsy samples were obtained, all of which showed invasive moderately differentiated SCC (**Figure 1B**). Immunohistochemistry (IHC) revealed moderately positive expression of P40 and CK5/6 in SCC cells (**Figures 1C, D**). In addition, the PD-L1 expression level in tumor cells was 50%, and the programmed cell death protein 1 (PD-1) expression level in immune cells was 3% (**Figures 1E, F**). IHC also showed high levels of expression of P16 and P63, which may be immunohistochemical markers of SCC in the cervix with metastasis to the duodenum. Contrast-enhanced CT of the abdomen showed a mass approximately 5.8 × 4.7 cm in size in the periampullary area (**Figure 2A**) and multiple enlarged retroperitoneal lymph nodes, the largest being approximately 1.0 cm in size (**Figure 2B**). 18 F-Fluorodeoxyglucose positron emission tomography/computed tomography (18F-FDG PET-CT) from the skull base to mid-thigh showed a periampullary lesion with a delayed-phase standardized uptake value (SUV) of 13.5, with metastasis to multiple lymph nodes around the duodenum, retroperitoneal para-aortic nodes, mesenteric root, left supraclavicular fossa, and left thoracic entrance area (delayed-phase SUV of 5.0). However, no abnormal hypermetabolic foci were found in the uterine body or cervix, and no cervical mass was found on contrast-enhanced CT of the chest, abdomen, and pelvis. Moreover, bimanual examination revealed no neoplastic lesions, and a thin-prep cytologic test (TCT) of cell samples obtained during a vaginal speculum examination revealed a low-grade squamous intraepithelial lesion in the epithelial cells of the cervix. After a multidisciplinary team discussion, the patient was diagnosed with primary SCC of the periampullary area with multiple lymph node metastases (stage IV).

**Figure 3:**
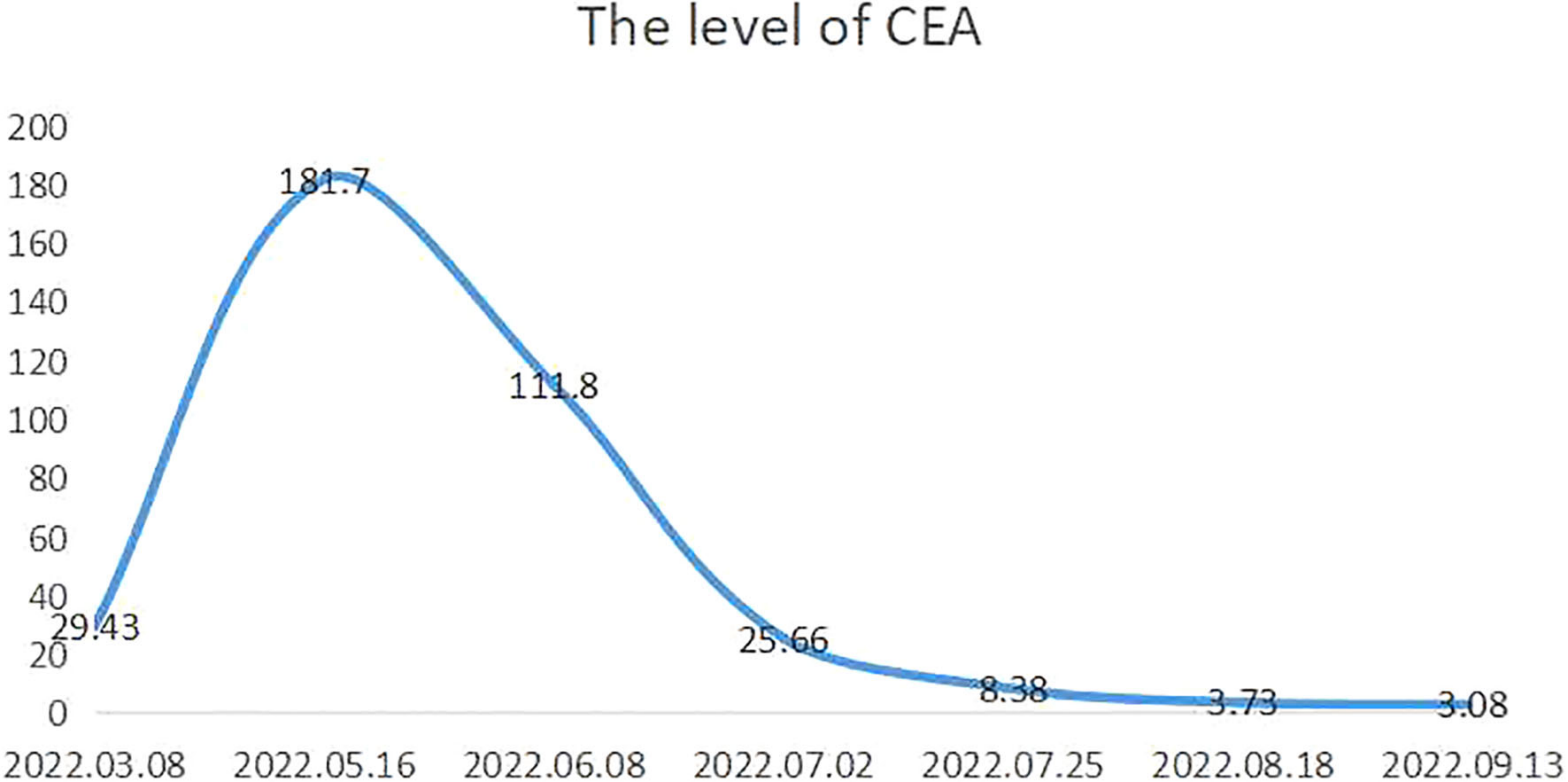
Carcinoembryonic antigen levels during treatment.

The patient received a cycle of albumin paclitaxel in combination with cisplatin but was not hospitalized. When she returned for further consultation 2 months later, her CEA level was clearly elevated. She received three weekly cycles of the original chemotherapy regimen in combination with immunotherapy (sintilimab 200 mg IVGTT D1) owing to upregulated PD-L1 expression. No complications were observed, and the most recent chest and abdominal enhanced CT indicated that the primary lesion (**Figure 2C**) and metastasized lymph nodes in the retroperitoneal space (**Figure 2D**) had shrunk (partial response). Moreover, CEA fell to 3.08 ng/ml (normal value: <5.0 ng/ml) (**Figure 3**).

## 3 Discussion

We report a case of advanced primary SCC of the periampullary area with upregulation of PD-L1 expression in tumor cells. Most metastatic SCCs of the periampullary area develop from other solid organs, such as the lung, cervix, larynx, or esophagus (3, 4). Primary squamous malignancy of the periampullary area is a rare pathology, with only occasional reported cases; a mass or ulcer in the periampullary area could be found during esophagogastroduodenoscopy. Kanthan et al. adopted CK5, p63, and p16 as immunohistochemical markers to confirm the diagnosis of SCC of the cervix with metastasis to the duodenum (5). In our case, we hold the view that SCC of the periampullary area was the primary tumor; this view was formed as a comprehensive judgment based on the results of PET-CT, pelvic contrast-enhanced CT, and TCT. Moreover, cervical cancer metastasis to the duodenum is rare, with only eight cases reported in the English-language literature since 1981. In two of these cases, there were synchronous metastases to the first part of the duodenum in stage IIIB or IV of SCC of the cervix at diagnosis (6). In other words, misdiagnosis of advanced cervical cancer with synchronous metastases by PET-CT is unlikely.

Periampullary tumors include tumors originating in the distal bile duct, pancreatic head, ampulla of Vater, and periampullary duodenum (7). They comprise four histologic epithelial types: ampullary, duodenal, pancreatic, and biliary (8). The origin of SCCs in the duodenum is probably associated with buried nests of ectopic squamous cells under the normal duodenal epithelial cells. Another hypothesis is that they originate from squamous metaplasia of multipotent stem cells in the basal layer of the duodenal mucosa, which then undergo malignant transformation to form SCC, according to several theories proposed in the literature (9, 10).

The optimal approach to treatment of advanced primary SCC of the periampullary area remains to be identified, since, thus far, sample sizes have been small and follow-up data are unavailable. Chemotherapy regimens including paclitaxel plus carboplatin and gemcitabine plus carboplatin have been mentioned in the literature in this context (11–13). To date, immune checkpoint inhibitors (ICIs) have been employed in immunotherapy for gastrointestinal SCC. Malignant cells can escape immune destruction by developing mechanisms regularly employed by the immune system for self-regulation. PD-L1 engages with the PD-1 receptor and induces PD-1 signaling, resulting in T-cell-mediated immunosuppression (14). Tumor cells can co-opt the PD-1 pathway to evade immune responses by expressing PD-1 ligands on the cell surface and engaging immune effector cells with PD-1 receptor expression. The predictive role of PD-L1 expression for PD-1/PD-L1 inhibitors has been reported in many types of cancers (15); upregulation of PD-L1 has also since been reported in various types of human cancers, including esophageal SCCs and immune cells, and several anti-PD1 monoclonal antibodies have shown marked and durable responses in several types of refractory tumors (16). In addition, Thakur et al. (1) have found that higher PD-L1 expression is significantly associated with improved survival in patients with periampullary cancer. Hence, we decided to pursue immunotherapy with sintilimab in this case; the patient benefited from this treatment, as the lesion shrank and CEA level fell significantly. In previously reported cases of SCC of the periampullary area, the PD-L1 expression level of the tumor has not been mentioned. As such, the level of expression of PD-L1 in tumor cells of SCC of the periampullary area should be measured to determine a potential approach to therapy based on immune targets.

## Data availability statement

The original contributions presented in the study are included in the article/supplementary material. Further inquiries can be directed to the corresponding authors.

## Ethics statement

The studies involving human participants were reviewed and approved by the Medical Research Ethics Committee of 900TH Hospital of Joint Logistics Support Force (approval No. 2022-024). The patients/participants provided their written informed consent to participate in this study. Written informed consent was obtained from the individual(s) for the publication of any potentially identifiable images or data included in this article.

## Author contributions

Concept and design: BW and DL. Article writing: BW and DL. Extraction and collection of data: BW and DZ. Drafting and revision of manuscript: BW, DL, CJ, and WW. Supervision: CJ and WW. Final approval of the manuscript: BW, DL, DZ, WW, and CJ. All authors contributed to the article and approved the submitted version.
